# Non-Coding RNAs in Adrenocortical Cancer: From Pathogenesis to Diagnosis

**DOI:** 10.3390/cancers12020461

**Published:** 2020-02-17

**Authors:** Abel Decmann, Pál Perge, Peter Istvan Turai, Attila Patócs, Peter Igaz

**Affiliations:** 1 2nd Department of Internal Medicine, Faculty of Medicine, Semmelweis University, Szentkirályi Str. 46., H-1088 Budapest, Hungary; decmann.abel@med.semmelweis-univ.hu (A.D.); paul.perge@gmail.com (P.P.); peteturai@gmail.com (P.I.T.); 2 MTA-SE Lendület Hereditary Endocrine Tumors Research Group, H-1089 Budapest, Hungary; patocs.attila@med.semmelweis-univ.hu; 3 Department of Laboratory Medicine, Semmelweis University, H-1089 Budapest, Hungary; 4 Department of Molecular Genetics, National Institute of Oncology, H-1122 Budapest, Hungary; 5 MTA-SE Molecular Medicine Research Group, H-1088 Budapest, Hungary

**Keywords:** non-coding RNA, microRNA, lncRNA, adrenocortical cancer, biomarker

## Abstract

Non-coding RNA molecules including microRNAs and long non-coding RNAs (lncRNA) have been implicated in the pathogenesis of several tumors and numerous data support their applicability in diagnosis as well. Despite recent advances, the pathogenesis of adrenocortical cancer still remains elusive and there are no reliable blood-borne markers of adrenocortical malignancy, either. Several findings show the potential applicability of microRNAs as biomarkers of malignancy and prognosis, and there are some data on lncRNA as well. In this review, we present a synopsis on the potential relevance of non-coding RNA molecules in adrenocortical pathogenesis and their applicability in diagnosis from tissue and blood.

## 1. Non-Coding RNA Molecules

Non-coding RNAs (ncRNA) are RNA molecules that do not encode proteins, but participate in the regulation or modulation of gene expression, thus they are considered to be parts of the epigenetic machinery. Small non-coding RNA molecules (sncRNA) are microRNAs (miRNA), short interfering RNAs (siRNA), and PIWI-interacting RNAs (piRNA). Among ncRNAs, miRNA studies are relatively abundant in comparison to other subclasses. ncRNAs that are longer than 200 nucleotides are called long non-coding RNAs (lncRNA). At least 200,000 unique lncRNAs are described in human tissues [1]. They can appear as linear or circular (circRNA) RNA molecules. Linear lncRNAs are important regulatory molecules at transcriptional and posttranscriptional levels [2]. circRNAs can act as microRNA sponges, thereby holding back miRNAs to bind to mRNAs and initiate degradation [3]. Moreover, these RNA molecules are able to regulate transcription and splicing, can inhibit ribosomal RNA maturation, and they can function as adaptors in altering protein-protein interaction [4]. miRNAs, lncRNAs, and circRNAs have tissue specific appearance and are relatively stable [3,5,6,7]. In this review, we present studies that investigate the role of ncRNAs in adrenocortical cancer pathogenesis, diagnosis and therapy. So far, only miRNAs and lncRNAs have been studied in this area, hence our review presents these findings.

## 2. MicroRNAs

MicroRNAs (miRNA) are short (19–25 nucleotides long) non-coding RNA molecules acting as epigenetic regulators of gene expression, mostly at the post-transcriptional level. miRNAs can silence messenger RNAs (mRNAs) as the endogenous mediators of RNA interference [8]. miRNAs are implicated in the regulation of several physiological and pathological processes including cell cycle, cell proliferation, apoptosis, immune regulation, etc. Dysregulation of miRNA expression is considered to be an early step of tumor formation [9,10]. miRNAs can function as oncogenes (oncogenic miRNAs, oncomiRs) and tumor suppressor miRNAs based on their classical oncogene-tumor suppressor dichotomy, and this can be related to their relative expression level, as overexpressed miRNA are considered to be oncogenic, but are also underexpressed as tumor suppressor [11].

miRNAs are expressed in a tissue-specific manner. One miRNA can target several different mRNAs and several different miRNAs can target a specific mRNA, thus miRNA actions are redundant and pleiotropic as well. The expression of miRNAs in tissues can thus be very different. A miRNA can have tumor suppressor function in one tissue and function as an oncogene in another [11].

## 3. MiRNAs in the Pathogenesis of Adrenocortical Cancer

Despite recent significant advances in the understanding of adrenocortical cancer (ACC) pathogenesis, we are still far from having a clear picture of its development. Several molecular events in signaling pathways were shown to have an impact on ACC tumorigenesis [12]. Germline and somatic mutations in the *TP53* (tumor protein p53) gene [13,14], activation of Wnt/ß-catenin pathway through somatic *CTNNB1* (catenin beta 1) mutations [15], overexpression of *IGF-2* (insulin-like growth factor 2) [16,17] and *SF1* (steroidogenic factor-1) genes [18], and mutations in mismatch repair genes [19] are all related to ACC formation. As miRNAs can target mRNAs, they are also supposed to be implicated in the pathogenesis of ACC. In the following, we present the available findings on the relevance of miRNAs in ACC pathogenesis.

### 3.1. Molecular Pathways Affected by miRNA in ACC

**Figure 1:**
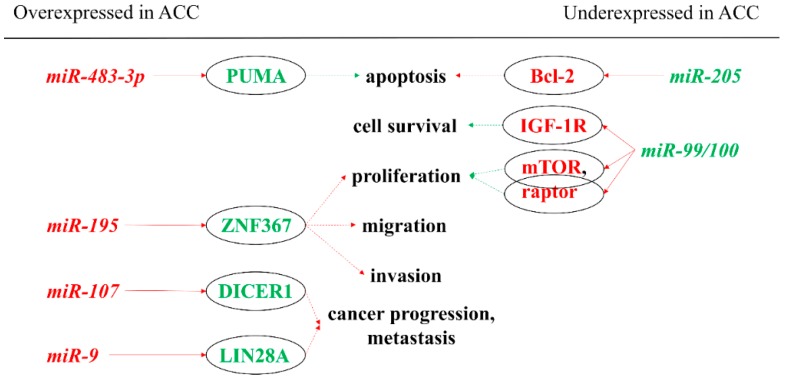
miRNAs showing oncogenic and tumor suppressor actions in the pathogenesis of ACC by targeting mRNAs. Red indicates inhibition, whereas green indicates stimulation. A solid arrow indicates a direct relationship, a dotted arrow indicates an indirect relationship. Red letters indicate oncogenic function; green letters indicate tumor suppressor function. ACC: adrenocortical cancer; Bcl-2: B-cell lymphoma 2; IGF-1R: insulin-like growth factor 1—receptor; miR: microRNA; mTOR: mammalian target of rapamycin; PUMA: p53 upregulated modulator of apoptosis; ZNF367: zinc finger protein 367.

First, we describe the associations of miRNAs to molecular pathways that are considered to be relevant in carcinogenesis, especially in the pathogenesis of ACC (Figure 1). From the previously listed genes, only *IGF-2* could be linked to miRNA-based regulation in ACC thus far. The *IGF-2* gene is known to be overexpressed in ACC, and the translated protein binds to the insulin-like growth factor 1 receptor (IGF-1R). The activation of this signaling pathway is thought to be relevant in adrenocortical tumorigenesis [20]. *miR-483-5p* and *miR-483-3p* are miRNAs found to be overexpressed in ACC by several studies [21,22,23,24,25]. Their genes are encoded in the intronic region of *IGF-*2 [26]. Simultaneously to *IGF-2* overexpression, levels of these miRNAs are also elevated. A significant positive correlation can be demonstrated between these two miRNAs and *IGF-2* expression [21,27]. On the other hand, two out of the most down-regulated miRNAs in ACC are *miR-99a* and *miR-100*. These miRNAs can bind to the *IGF-1R* and *mTOR* (mammalian target of rapamycin) mRNAs thereby regulating the expression of these proteins [28]. However, the underexpression of *miR-100* could not be correlated with *IGF-1R* expression in ACC [29].

Silencing *miR-483-3p* in the hepatocellular carcinoma cell line (HepG2) resulted in decreased cell proliferation and increased apoptosis [26]. A similar phenomenon was observed in the human ACC cell line (NCI-H295R) as well [22]. *miR-483-3p* was validated to target the pro-apoptotic *PUMA* (p53 upregulated modulator of apoptosis) mRNA, and a significant negative correlation was found between the expression of its protein product and *miR-483-3p* in Wilms’ tumor and other common tumors (colon, breast, liver) [26]. *miR-483-5p* was found to promote cell proliferation in the NCI-H295R cell line, but in contrast to *miR-483-3p*, it had no effect on apoptosis (PUMA was not found to be a target of *miR-483-5p*) [22].

A number of zinc finger proteins are involved in cancer progression [30]. Zinc finger protein 367 (*ZNF367*) is overexpressed in ACC compared to benign adrenal adenomas. This protein was found to have effects on cellular adhesion, invasion, migration, and adhesion [31]. It was also demonstrated that *miR-195* can directly target *ZNF367* and thus regulate cellular invasion. A negative correlation between the expression of *miR-195* and the ZNF367 protein was found [31].

Some advanced and poorly differentiated cancers display *LIN28A* and *LIN28B* overexpression [32,33]. Significant overexpression of *LIN28A* was observed in ACC relative to adrenocortical adenoma (ACA). In aggressive ACC compared to its non-aggressive counterpart, *LIN28* protein expression was low, but a negative regulator of this protein, *miR-9*, was overexpressed. This miRNA showed strong correlation with survival in ACCs. *miR-9* was also overexpressed in ACC compared to ACA and normal adrenal cortices [34].

The dysregulation of *DICER1* that is a principal enzyme in miRNA maturation is also observed in a number of various cancers [35,36], while *miR-103/miR-107* family has been shown to regulate the expression of *DICER1* [37]. *DICER1* is significantly overexpressed in ACC compared to ACA. Among ACC, low expression of *DICER1* associated with poor survival and may serve as a prognostic factor for local recurrence or metastases. In ACC, *miR-107* was overexpressed relative to ACAs [38].

### 3.2. Potential Pathogenic Relevance of miRNA in ACC by High Throughput Techniques

As a second approach, we present data obtained using high throughput techniques to compare the global miRNA expression profiles of ACC and ACA or normal adrenal cortices, in order to find differentially expressed miRNAs. MiRNAs showing the largest differences are then subjected to target prediction or pathway analysis to identify the most relevant pathomechanisms, and then some of these are validated.

In our first study on the expression of miRNAs in adrenocortical tumors, we have found significantly differentially expressed miRNAs in ACC compared to ACA and normal adrenals including overexpressed *miR-503*, *miR-184*, *miR-210*, *miR-214*, *miR-375*, and *miR-511* in ACC. Complex pathway analysis of these miRNAs revealed a cell cycle damage at G2/M checkpoint in ACC [39,40].

Schmitz et al. identified three significantly down-regulated miRNAs in ACC compared to ACA. Pathway analysis of *miR-139*, *miR-675*, and *miR-335* revealed that miRNAs’ targets include zinc finger proteins (Krueppel-related zinc finger protein 4: *GLI4* and PR domain zinc finger protein 2: *PRDM2*) [41].

Underexpressed *miR-205* was found in ACC compared to ACA by Wu et al. Overexpression of *miR-205* could induce apoptosis, block proliferation in cell culture (SW-13 adrenocortical cancer cell line) and also inhibit in vivo tumor growth, suggesting a tumor suppressor role for this miRNA. Target prediction revealed *Bcl-2* (B-cell lymphoma 2) as a potential target of *miR-205*. In SW-13 cells, a negative correlation between the miRNA and *Bcl-2* expression could be observed. *miR-205* can therefore potentially inhibit tumor progression in ACC via targeting *Bcl-2* [42].

## 4. MiRNAs as Potential Biomarkers

MiRNAs can be exploited as biomarkers since they are expressed in a tissue-specific fashion, and their expression is altered due to various diseases, including tumors. Furthermore, miRNAs are rather stable, thereby enabling their retrieval from formalin-fixed paraffin-embedded (FFPE) samples and also from body fluids. To find miRNA biomarkers, tissue miRNA expression profiles have to be compared between diseases (Table 1). Three main discovery methods are used to differentiate between groups (polymerase chain reaction (PCR), microarray, and next generation sequencing (NGS)). PCR-based and microarray studies can only investigate already known miRNAs, while using NGS can also enable previously unknown non-coding miRNAs to be identified.

Some studies applied snap frozen tissues, while others used formalin-fixed paraffin-embedded tissues. miRNA expression in FFPE samples are in correlation with those from snap frozen samples [43]. Formalin fixation does not affect the miRNA expression levels, in contrast to those of mRNA and DNA [43]. The superiority of any starting material has not been yet determined.

To correctly compare miRNA expression profiles in samples, pre-analytical variations should be considered. To avoid the imprecisions from these variations, normalization or standardization of samples is necessary. For this purpose, “housekeeping” RNAs that have similar expression in different samples, or externally added controls (like the C. elegans cel-miR-39) are used. A number of such molecules have been identified and used so far, but there is no consensus on which reference should be used for miRNA studies [44,45,46]. This represents a major problem with the analysis of miRNA expression, especially when body fluids with low miRNA content are used as the source material.

One of the first studies to determine the tissue miRNA expression profile of adrenocortical tumors was performed by our research group (Tömböl et al., [39]). Significantly up-regulated (*miR-503*, *miR-210*, and *miR-184*) and down-regulated (*miR-511*, *miR-214*, and *miR-375*) tissue miRNAs were found in ACC compared to ACA samples. The most overexpressed miRNA was *miR-503* and the most down-regulated was *miR-511.* When using the difference between these two miRNAs as diagnostic marker in the differentiation of ACC and ACA, a sensitivity of 100% and specificity of 97% could be achieved (Table 2). This study, however, included only a small number of ACCs (*n* = 7) [39]. In the study of Feinmesser et al., *miR-503* was also found to be significantly overexpressed in ACCs compared to ACAs, and was found to have perfect diagnostic accuracy, with values of 100% sensitivity and specificity [47]. Furthermore, this miRNA might have a prognostic role, as was reported by Özata et al., while higher expression of *miR-503* was associated with poorer prognosis in ACCs [22].

**Table 1:** Studies on tissue miRNA expression profiling in ACC and ACA.

Discovery Method	Discovery Cohort (*n*)	Validation Method	Validation Cohort (*n*)	Sample Type	Expression in ACC vs. ACA	Reference
Microarray	22 ACC, 27 ACA, 6 NA	RT-qPCR-	9(5) ACA, 10(6) ACC	Snap frozen	↓ miR-195, miR-335	[48]
Microarray	4 ACC, 8 ACA, 4 NA	RT-qPCR	7(3) ACC, 19(11) ACA, 10(6) NA	Snap frozen	↓ miR-214, miR-511, miR-375; ↑ miR-503, miR-184, miR-210	[39]
Microarray	7 ACC, 9 APA, 4 NA	RT-qPCR	15 AT	FFPE	↓ miR-139-3p, miR-335, miR-675	[41]
Microarray	10 ACC, 26 ACA	RT-qPCR	29 ACC, 35 ACA	Snap frozen	↓ miR-195, miR-125b, miR-100; ↑ miR-483-5p	[21]
Microarray	22 ACC, 26 ACA, 4 NA	RT-qPCR	25(3) ACC, 43(17) ACA, 10(6) NA	Snap frozen	↓ miR-1974, miR-195, miR-497; ↑ miR-483-3p, miR-483-5p, miR-210, miR-21	[22]
Microarray	6 ACC, 6 ACA, 6 NA	RT-qPCR	18 ACC, 10 ACA, 3 NA	Snap frozen	↓ miR-335, miR-195; ↑ miR-139-5p, miR-376a, miR-376b, miR-376c, miR-483-5p	[24]
NGS	45 ACC, 3 NA			Snap frozen	↓ miR-195, miR-335; ↑ miR-483-3p, miR-483-5p, miR-210, miR-503	[25]
qRT-PCR	51 ACC, 47 ACA			FFPE	↓ miR-195; ↑ miR-483-3p, miR-483-5p, miR-210	[23]
Microarray	8 ACC, 25 ACA	RT-qPCR	11 ACC, 4 ACA,	FFPE	↓ miR-335, miR-195, miR-497; ↑ miR-503	[47]
Microarray	10 ACC, 26 ACA, 21 NA			Snap frozen	↑ miR-9, miR-25, miR-124, miR-183, miR-185, miR-206	[49]
NGS	7 ACC, 7 ACA, 8 NA	RT-qPCR	8 ACC, 8 ACA, 9 NA	FFPE	↑ miR-503, miR-483-5p, miR-450a, miR-210, miR-483-5p, miR-421	[50]
NGS	10 ACC, 10 ACA, 10 AML	RT-qPCR	12 ACC, 14 ACA, 15 AML	FFPE	↑ miR-184, miR-483-5p, miR-483-3p, miR-183-5p	[51]

ACA: adrenocortical adenoma; ACC: adrenocortical carcinoma; AML: adrenal myelolipoma; AT: adrenal tumor; FFPE: formalin-fixed paraffin-embedded; miR: microRNA; NGS: next-generation sequencing; NA: normal adrenal; qRT-PCR: quantitative reverse-transcription polymerase chain reaction.

In a larger cohort, Soon et al. have found significantly down-regulated *miR-195* and *miR-335* in ACCs compared to ACAs. *miR-7* was found to be significantly underexpressed in both ACA and ACC compared to normal adrenals [48]. Another study found *miR-139*, *miR-335*, and *miR-675* to be down-regulated in ACC relative to ACA [41]. Further down-regulated tissue miRNAs in ACC were *miR-100*, *miR-125b*, and *miR-195*, while and up-regulated *miR-483-5p* could discriminate between ACC and ACA with a sensitivity and specificity of 80% and 100%, respectively [21]. Özata et al. confirmed the up-regulation of *miR-483-5p* together with *miR-483-3p*, *miR-210*, and *miR-21* in ACCs. *miR-210* is considered to be a general hypoxamiR, as its expression is observed in a wide array of tumors displaying a hypoxic environment [52]. On the other hand, miR-21 is overexpressed in many different tumors, and could be regarded as a wide-spread oncogenic molecule [53]. Unfortunately, there are no mechanistic studies yet showing the relevance of miR-210 and miR-21 in ACC tumorigenesis. Down-regulation of *miR-1974*, *miR-195*, and *miR-497* was also observed in ACC compared to ACA [22]. Similar observations of differentially expressed miRNAs were done by Duregon et al. without validation [23]. *miR-335* and *miR-195* were significantly underexpressed in ACC, while *miR-139*, *miR-376a*, *-b*, *-c*, and *miR-483-5p* were significantly up-regulated in ACCs compared to ACAs [24]. Assié et al. found down-regulated *miR-195* and *miR-335*, whereas *miR-483-3p*, *miR-483-5p*, *miR-210* and *miR-503* were up-regulated in ACC compared to normal adrenal cortices. These results are not validated in independent cohorts, however, they support former studies [25].

An integrated genome-wide analysis has shown that *miR-9*, *miR-25*, *miR-124*, *miR-183*, *miR-185*, and *miR-206* were overexpressed in ACC compared to ACA and were associated with down-regulated gene expression of at least 10 genes in ACC [49].

miRNAs can be useful not only for the differentiation of tumors, but they can also have prognostic relevance. *miR-483-5p*, *miR-195*, *miR-503*, *miR-1202*, and *miR-1275* miRNAs were significantly associated with poor prognosis of ACC [22,48].

In a recent study on tissue miRNA, Koperski et al. found significantly up-regulated miRNAs (*miR-503-5p*, *miR-483-3p*, *miR-483-5p*, *miR-210*, *miR-450a*, *miR-421*) that in part corresponded to those mentioned previously, in ACC compared to ACA. Diagnostic applicability of significantly differentially expressed miRNAs was evaluated by using ROC-analysis. Area under curve was 100% only for *miR-503-5p*, however, the authors have recommended *miR-483-3p*, *miR-483-5p*, and *miR-210* in the molecular testing of ACCs, because these miRNAs have had higher mean expression than the others [50].

Despite many similarities between these different studies, several discrepant results can also be observed. Such discrepancies are general features of studies involving miRNA, as no standard protocols for sample retrieval, analysis and data interpretation are available yet. This problem is even more serious with the study of extracellular miRNA discussed in the next section.

## 5. Circulating miRNA as Biomarkers in ACC

Apart from tissues, miRNAs were also shown to be present in body fluids and thus can be exploited as minimally invasive (or even non-invasive) biomarkers. miRNAs are detectable in wide range of body fluids including plasma, saliva, tears, urine, amniotic fluid, colostrum, breast milk, bronchial lavage, cerebrospinal fluid, peritoneal fluid, pleural fluid, and seminal fluid [54]. The blood-derived miRNAs are called circulating miRNAs [55,56,57].

According to our present knowledge, the release of extracellular miRNAs from the parent cell can occur in three major ways [58]. Passive release due to cellular damage (necrosis, inflammation), or active secretion packed into extracellular vesicles (EV) (microvesicles, exosomes and apoptotic bodies) or in association with macromolecular complexes such as Argonaute (AGO) proteins (mainly AGO2) and high density lipoprotein (HDL) [59,60]. The vast majority (approximately 90%-95%) of the extracellular miRNAs are in complexes with AGO [61,62].

Based on novel experimental results, extracellular miRNAs in EVs and associated with HDL might be able to get into another cell and alter its gene expression [63,64]. Therefore, circulating miRNAs might act as hormone-like molecules by influencing the gene expression, even in distant cells or tissues [58,65,66]. In contrast, conflicting results assumed that extracellular miRNAs should be regarded as cellular byproducts (debris) lacking biological activity [67,68].

There have been eight studies investigating the pattern of extracellular miRNAs in adrenocortical tumors (ACT) to date [24,51,69,70,71,72,73,74] (Table 3).

All studies confirmed the significant overexpression of *miR-483-5p* in ACC versus ACA. Moreover, Patel et al. found the overrepresentation of *miR-34a* in ACC samples [69]. On the other hand, in the study of Chabre et al., the underrepresentation of *miR-195* and *miR-335* were confirmed in the ACC group [24]. Furthermore, they found a correlation between the expression of *miR-483-5p* and the aggressiveness of ACC. They detected *miR-483-5p* only in the serum of patients with aggressive ACC. Shorter overall and shorter recurrence-free survival were associated with increased *miR-483-5p* and decreased *miR-195* expression in the circulation [24]. In our previous study, we found significant alteration of blood-borne miRNAs isolated from whole plasma in ACT [70]. Five miRNAs (*miR-100*, *miR-181b*, *miR-184*, *miR-210*, and *miR-483-5p*) were significantly overexpressed in the plasma of patients suffering from ACC versus ACA. In a further study, the absolute plasma level of *miR-483* and *miR-483-5p* was measured, and a statistically significant overexpression was identified in advanced stages of ACC (stage III-IV) versus to local malignancy (stage I-II), to ACA and to healthy patients [71]. Moreover, a correlation between the level of *miR-483-5p* and the number of circulating tumor cells was also revealed.

According to our current knowledge, there is no unequivocal recommendation whether plasma or serum is more appropriate for the evaluation of extracellular miRNAs [56,75]. By applying serum, the extracted RNA yield could be higher, but the expression of miRNAs could be affected by the coagulation process [76]. In contrast by using plasma, the aforementioned process has no effect, but platelet contamination can easily occur [77].

Despite the promising results of the above studies, the sensitivity and specificity values of miRNA markers were variable. We hypothesized that by studying EV-associated miRNAs, the sensitivity and specificity of circulating miRNA could be enhanced. Since the active release of miRNAs to EVs seems to be a controlled process, EV-associated miRNAs could be more specific markers of malignancy [78]. We therefore investigated the expression of EV-associated miRNAs and their diagnostic applicability and found two miRNAs significantly overexpressed in ACC compared to ACA (*miR-101* and *miR-483-5p)* [72]. The diagnostic accuracy of EV-associated *miR-483-5p* was higher than in previous studies on unfractionated plasma or serum (87.5 % and 94.4 %, respectively, area under curve: 0,965) [72]. (Table 4) Therefore, EV-associated *miR-483-5p* appears to be a promising minimally invasive biomarker of ACC. The expression of *miR-483-5p* was not influenced by dynamic hormonal tests (low dose overnight dexamethasone and adrenocorticotropin stimulation) used in routine hormonal diagnostics that support its utility as a biomarker of ACC [79]. Moreover, circulating *miR-483-5p* could be applied as a marker of treatment efficacy too [80]. The expression of *miR-483-5p* was significantly decreased by a combined therapy (9-cis-retinoic acid and mitotane) in a mouse NCI-H295R xenograft model [80]. Regarding another circulating miRNA as a potential marker of ACC treatment efficacy, in a SW-13 xenograft model, significant changes in the expression of circulating *miR-210* (a major hypoxamiR, usually overexpressed under hypoxic conditions characteristic for malignant tumors) were noted by liposomal etoposide- doxorubicin-cisplatin-mitotane combined chemotherapy [81]. We have also found overexpression of circulating *miR-210* in cortisol-producing ACC [72].

In another recent study, we have compared the diagnostic applicability of urinary and whole plasma derived miR-483-5p [74]. Significant overexpression of whole plasma miR-483-5p was again confirmed in ACC relative to ACA, but although despite being detectable in the urine, no difference in urinary miR-483-5p was found between ACA and ACC.

Despite these promising findings underlining the clinical applicability of *miR-483-5p*, our recent study has revealed a potential limitation [51]. The expression of tissue miRNAs and their circulating counterparts were evaluated in patients with adrenal myelolipoma (AML), ACA and ACC, and no significant difference was found between the expression of *miR-483-3p* and *miR-483-5p* in AML and ACC samples that might limit the clinical applicability of *miR-483-5p*. On the other hand, *miR-451a* could be a promising biomarker for AML.

We have also studied the expression of circulating miRNAs in hormonally inactive and cortisol-producing ACTs [73]. *miR-22-3p*, *miR-27a-3p*, and *miR-320b* were significantly overexpressed in both cortisol-producing ACA (CPA) and ACC (CP-ACC) compared to non-functioning ACA (NFA). The expression of *miR-210-3p* was significantly increased only in CP-ACC compared to NFA. Moreover, significant correlations were revealed between the 24-h urinary free cortisol level and the expression of *miR-22-3p*, *miR-27a-3p*, and *miR-320b*. In addition, correlations between the cortisol level after low dose dexamethasone test and EV-associated miRNA expression were also established. In our previous study, we demonstrated that circulating whole plasma derived *miR-27a* is influenced by dexamethasone and adrenocorticotropin both in vivo and in vitro [79]. In this study, we have confirmed that EV-associated *miR-27a* was also induced by dexamethasone. Based on these results, it can be supposed that hypercortisolism might have a role in the overexpression of these miRNAs.

It must be noted that despite these promising results, there were significant differences in the results of studies performed on circulating miRNA. The different methodology, the diversity of the reference genes, and the relative low number of patients involved could contribute to this discrepancy. Further studies on larger cohorts with uniform methodological requirements are warranted to clarify the applicability of circulating miRNA as biomarkers of malignancy, prognosis and follow-up. On the other hand, in contrast to their tissue counterparts, there are no data on the biological relevance of circulating miRNA in adrenocortical tumors [82,83]. miRNA might work well for differentiating two disease entities (e.g., *miR-483-5p* for differentiating adrenocortical adenoma and cancer), but due to their wide-spread expression in other organs, the specificity of miRNA for a given disease could be limited.

## 6. miRNAs as Potential Therapeutic Mediators or Targets

miRNAs as major regulators of gene expression are considered to be promising candidates for targeted molecular therapies. Soon et al. have shown that *miR-7* was significantly down-regulated in ACCs compared to normal adrenals [48]. The next study conducted by the same group aimed to find a potential miRNA therapeutics for ACC. When reinstating *miR-7* expression, it was observed that this miRNA negatively affects cell proliferation and induces arrest in the G1 phase of cell cycle in cell culture and reduced tumor growth in ACC xenograft mouse model (H295R). The direct targets of *miR-7* are the *MAPK* (mitogen-activated protein kinase) and *mTOR* signaling pathways, which lead to inhibition of *CDK1* (cyclin dependent kinase 1) [84].

In a further study, the expression and targets of *miR-483-5p* and *miR-193-5p* were investigated in ACC. The expression level of the two miRNAs correlated with the aggressiveness of ACC. The most down-regulated potential target gene of *miR-483-5p* was the N-myc downstream-regulated gene 2 (*NDRG2)*, whereas the most downregulated target gene for *miR-139-5p* was *NDRG4*. The expression of these two genes was negatively correlated with the aggressiveness of ACC. Therefore, these genes were suggested to be tumor suppressor genes. When the expression of these two miRNAs was inhibited in cell lines, the two target genes were overexpressed. The inhibition of *miR-483-5p* and *miR-139-5p* and the restoration of the two genes suppressed the invasive potential of ACC cell lines in vitro [85].

An additional miRNA, *miR-497*, was found to be significantly down-regulated in ACC and could post-transcriptionally repress the long non-coding RNA (lncRNA) *MALAT1*. A molecular target of *miR-497* is the eukaryotic translation initiation factor 4E (*iIF4E*). *MALAT1* also competes for the binding site of this miRNA. It was demonstrated that the overexpression of *miR-497* and the silencing of *MALAT1* down-regulates *eIF4E*, and thus suppress cellular proliferation and induces cell cycle arrest [86].

## 7. Long Non-Coding RNA Molecules in ACC

Long non-coding RNAs (lncRNA) are at least 200 nucleotides long and do not encode proteins [87]. lncRNAs are generally stable, but similarly to mRNAs exhibit a broad range of variation in their stability profile, making them a dynamic component in response to environmental changes that can modulate gene expression. On the other hand, highly stable lncRNAs may play a role in housekeeping functions [88,89]. Their stability depends upon their genomic position, subcellular localization, RNA binding proteins, splicing, and guanine-cytosine content, but their stability is not correlated with their rate of expression [89]. lncRNAs are thought to encompass nearly 30,000 different transcripts in humans, hence lncRNA transcripts account for the major part of the noncoding transcriptome. Long noncoding RNAs play a significant role in carcinogenesis. lncRNAs control various biological processes via multiple mechanisms, including guiding epigenetic modifiers and transcription factors to their target genes, functioning as sponges for endogenous RNAs, regulating mRNA decay, mediating chromosomal interactions, etc. Furthermore, lncRNAs exhibit higher specificity in expression profile than mRNAs not only in cell, tissue, and developmental stages, but also in a disease state-specific manner [87].

In contrast to the several studies on microRNAs conducted in adrenocortical tumors, there has only been one study reporting on the expression of long noncoding RNAs (lncRNA) so far [48,90,91,92]. This study sheds light on differential expression patterns of lncRNAs in adrenocortical carcinoma (ACC), adrenocortical adenoma (ACA), as well as in normal adrenal cortex (NAC) and suggests novel prognostic markers and therapeutic targets [93].

Both microarray and next generation sequencing are appropriate methods for assessing lncRNA expression [94], but there are reports preferring microarray [95]. The study on ACC used a microarray platform [93].

In the ACC-NAC comparison, 476 lncRNAs were up-regulated and 480 were down-regulated in ACC [93]. GAS5 (growth specific arrest 5), a tumorsuppressor lncRNA in breast cancer was also found to be downregulated in ACC [96]. Other examples for cancer-related, differentially expressed lncRNAs were guanine nucleotide-binding protein, *GNAS-AS1* (alpha-stimulating-antisense 1), *H19*, *MALAT1* (metastasis-associated lung adenocarcinoma transcript 1), *PRINS* (psoriasis-associated RNA induced by stress) and maternally expressed 3 (*MEG3*) [97,98].

Regarding the ACA NAC comparison, 1999 lncRNAs were found to be upregulated, whilst 656 lncRNAs were downregulated in ACC. Interestingly, the cancer-related MALAT1 was one of the most upregulated lncRNA [99]. In recurring ACC, only *PRINS* has been proved to be significantly associated with non-recurring ACC, and this lncRNA appears to be a relevant tumor suppressor in ACCs [93].

Also, *PRINS* was found to be down-regulated in metastatic ACC. PRINS will be worthy of further studies and it might be exploited as a prognostic marker or even as a therapeutic target. Despite these findings on the potential tumor suppressor activity of PRINS, its expression could not be correlated to overall survival in ACC [93].

## 8. Conclusions

According to the latest findings, non-coding RNAs are of great importance regarding not only in physiological processes, but also in the pathogenesis of various diseases. Both miRNAs and lncRNAs are able to function as tumor suppressors or oncogenes, and their aberrant expression can lead to tumorigenesis. In the previous two decades, we have learnt a lot about miRNAs. The pathogenesis of adrenocortical cancer is still poorly elucidated, and studies on non-coding RNA can contribute to its better understanding. A very promising field of ncRNA (mostly miRNA) in ACC is related to their use as possible biomarkers not only in tissues, but also as minimally invasive biomarkers as a form of liquid biopsy from biofluid samples. Moreover, ncRNAs are also potential candidates for genetic therapies in oncological care.

## Figures and Tables

**Table 2:** Diagnostic accuracy of differentially expressed miRNAs in tissue samples.

miRNA	AUC	Sensitivity/Specificity	Reference
*miR-511*	0.985	100%/93%	[39]
Difference of *miR-511* and *miR-503*	0.995	100%/97%	[39]
Difference of *miR-511* and *miR-184*	0.970	100%/80%	[39]
*miR-34* and *miR-497*	n.a.	100%/96%, 100%/89%	[47]
*miR-100*	0.717	n.a.	[21]
*miR-125b*	0.763	n.a.	[21]
*miR-195*	0.771	n.a.	[21]
*miR-195*	0.830	69%/94%	[24]
*miR-210*	1.000	n.a.	[50]
*miR-335*	0.877	88%/88%	[24]
*miR-421*	0.954	n.a.	[50]
*miR-450a-5p*	0.974	n.a.	[50]
*miR-483-3p*	0.987	n.a.	[50]
*miR-483-5p*	0.943	100%/80%	[21]
*miR-483-5p*	0.904	74%/100%	[24]
*miR-483-5p*	1.000	n.a.	[50]
*miR-503*	n.a.	100%/100%	[47]
*miR-503-5p*	1.000	n.a.	[50]

Diagnostic accuracy was assessed by receiver operating characteristics (ROC) analysis in most of the studies. Available sensitivity and specificity values are presented in the table. AUC: are under curve; miR, miRNA: microRNA; n.a.: not available.

**Table 3:** Summary of studies reporting circulating miRNA expression findings in adrenocortical cancer.

Author and Year of Publication	Reference	Source	Method	Samples	miRNA Overexpressed in ACC	miRNA Underexpressed in ACC
Chabre et al. (2013)	[24]	Serum	qRT-PCR	23 ACC, 4 ACA, 19 NA	*miR-483-5p*	*miR-195*, *miR-335*
Patel et al. (2013)	[69]	Serum	qRT-PCR	17 ACC, 22 ACA	*miR-34a*, *miR-483-5p*	
Szabó et al. (2014)	[70]	Plasma	qRT-PCR	13 ACC, 12 ACA	*miR-100*, *miR-181b*, *miR-184*, *miR-210*, *miR-483-5p*	
Perge et al. (2017)	[72]	Plasma-extracellular vesicle	qRT-PCR	22 ACC, 24 ACA	*miR-101*, *miR-483-5p*	
Salvianti et al. (2017)	[71]	Plasma	qRT-PCR	27ACC, 13ACA, 10 NA	*miR-483-5p*	
Perge et al. (2018)	[73]	Plasma-extracellular vesicle	qRT-PCR	9 CP-ACC, 13 CPA, 13 NFA	*miR-22-3p*, *miR-27a-3p*, *miR-320b*, *miR-210-3p*	
Decmann et al. (2019)	[74]	Plasma	qRT-PCR	23 ACC, 23 ACA	*miR-483-5p*	
Decmann et al. (2018)	[51]	Plasma	qRT-PCR	11 ACC, 11 ACA, 11 AML	*miR-483-3p*, *miR-483-5p*	

Abbreviations: ACC: adrenocortical cancer, ACA: adrenocortical adenoma, AML: adrenal myelolipoma, CP-ACC: cortisol-producing ACC, NA: normal adrenal.

**Table 4:** Diagnostic applicability of extracellular microRNAs for the differentiation of adrenocortical cancer and adrenocortical adenoma.

Author and Year	Reference	Comparison	miRNA	Sensitivity	Specificity	AUC
Chabre et al. (2013)	[24]	ACC-**ACA**	*miR-195*	90.9	100	0.948
		**aACC**-naACC	*miR-139-5p*	87.5	65	0.714
		ACC-**ACA**	*miR-335*	95.2	71.4	0.837
		**aACC**-naACC	*miR-376a*	71.4	85.7	0.811
		**aACC**-naACC	*miR-483-5p*	85.7	100	0.929
Patel et al. (2013)	[69]	**ACC**-ACA	*miR-34a*	ND	ND	0.81
		**ACC**-ACA	*miR-483-5p*	ND	ND	0.74
Szabó et al. (2014)	[70]	**ACC**-ACA	*dCTmiR-210—dCTmiR-181b*	88.9	75	0.87
		**ACC**-ACA	*dCTmiR-100/dCTmiR-181b*	77.8	100	0.85
Perge et al. (2017)	[72]	**ACC**-ACA	*miR-483-5p*	87.5	94.44	0.965
		**ACC**-ACA	*miR-101*	68.75	83.33	0.766
Salvianti et al. (2017)	[71]		*miR-483*	87.5	63.6	0.875
			*miR-483-5p*	83.3	100	0.917
Perge et al. (2018)	[73]	**CP-ACC**-CPA	*miR-320b*	88.89	76.92	0.863
Decmann et al. (2018)	[51]	**ACC**-ACA	*miR-483-5p*	87	78.3	0.88
Decmann et al. (2019)	[74]	**ACC**-ACA	*miR-483-3p*	81.82	90.91	0.88

Abbreviations: AUC: area under curve, ACC: adrenocortical cancer, aACC: aggressive adrenocortical cancer, naACC: non-aggressive adrenocortical cancer, ACA: adrenocortical adenoma, ND: No data, CP-ACC: cortisol-producing ACC, CPA: cortisol-producing ACA.
